# Synergistic Paradigms in Infection Control: A Review on Photodynamic Therapy as an Adjunctive Strategy to Antibiotics

**DOI:** 10.1021/acsinfecdis.5c00369

**Published:** 2025-09-22

**Authors:** Jennifer Machado Soares, Thaila Quatrini Corrêa, Claudia Patricia Barrera Patiño, Isabella Salgado Gonçalves, Gabriel Grube dos Santos, Gabriela Gomes Guimarães, Rebeca Vieira de Lima, Thalita Hellen Nunes Lima, Bruna Carolina Corrêa, Taina Cruz de Souza Cappellini, Maria Vitória Silva Pereira, Anna Luiza França de Oliveira Resende, Vladislav V. Yakovlev, Kate Cristina Blanco, Vanderlei Salvador Bagnato

**Affiliations:** † São Carlos Institute of Physics, University of São Paulo, São Paulo 05508-900, Brazil; ‡ PPGBiotec, Federal University of São Carlos, São Carlos 13565-905, Brazil; § Biomedical Engineering, 14736 Texas A&M University, CPRIT Scholar in Cancer Research, College Station, Texas 77843, United States

**Keywords:** photodynamic therapy, multidrug-resistance, synergistic effect, antimicrobial strategies, reactive oxygen species

## Abstract

The increasing threat of antimicrobial resistance necessitates developing novel strategies to enhance the efficacy of existing antibiotics. This review explores the potential of antimicrobial photodynamic therapy (aPDT) as an adjunctive approach to antibiotic therapy. A systematic literature search was conducted in major scientific databases, focusing on studies published in the past decade investigating the synergistic effects of aPDT with antibiotics. Selected articles were analyzed based on their experimental approaches, bacterial targets, photodynamic parameters, and reported treatment outcomes. aPDT induces bacterial cell damage by generating reactive oxygen species (ROS), enhancing antibiotic susceptibility, and reducing required dosages. Furthermore, the review highlights promising research on optimizing treatment parameters and antibiotic combination strategies to maximize therapeutic outcomes. Despite its potential, aPDT faces obstacles to treatment standardization, variability in bacterial responses, and clinical implementation hurdles. These challenges require standardized protocols, further in vivo studies, and regulatory advancements to integrate aPDT into mainstream antimicrobial therapy. Conclusion: The synergy between aPDT and antibiotics represents a promising frontier in infection control, offering a safer, more effective, and resistance-mitigating strategy for bacterial infections. Future research should focus on refining treatment parameters, assessing long-term clinical impacts, and facilitating the widespread adoption of aPDT as a complementary antimicrobial approach.

## Graphical abstract

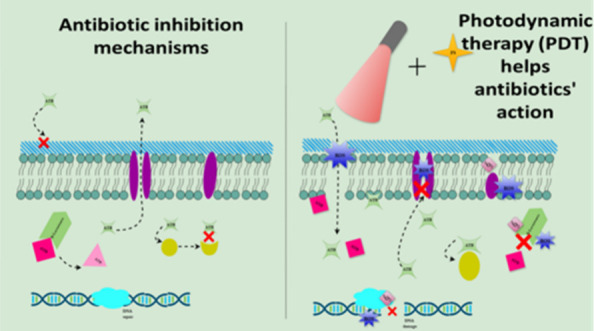

The rapid emergence of antimicrobial resistance (AMR) has become a critical global health concern, demanding urgent strategies to enhance the efficacy of existing antibiotics.^1^ The widespread misuse and overuse of antibiotics in human health, agriculture, and veterinary medicine have accelerated the selection of resistant microbial strains, leading to increased morbidity and mortality worldwide.^2^ The World Health Organization (WHO) has repeatedly warned that, without effective interventions, AMR could become one of the leading causes of death globally.^2,3^ The exhaustion of conventional antibiotic options and the slow pace of new antibiotic discovery underscore the urgent need for alternative and adjunctive approaches to antimicrobial therapy.

Among the promising strategies under investigation, antimicrobial photodynamic therapy (aPDT) has emerged as a potent adjunctive modality to conventional antibiotics, offering a novel mechanism to combat resistant pathogens.^4,5^ The core principle of aPDT relies on activating a photosensitizing agent (PS) by specific wavelengths of light, leading to the production of reactive oxygen species (ROS), which induce microbial destruction. Unlike traditional antibiotics, which target specific bacterial structures or metabolic pathways, aPDT operates through oxidative stress-mediated damage, reducing the likelihood of resistance development.^6^ Recent studies have demonstrated that aPDT exhibits broad-spectrum antimicrobial activity and enhances bacterial susceptibility to antibiotics, lowering the required antibiotic dose, reducing side effects, and minimizing selective pressure for resistance.^7–12^

The growing crisis of microbial resistance has been predicted for decades, with early warnings from researchers and public health organizations.^3,13,14^ They have evolved to develop resistance against both natural and synthetic antimicrobials, but modern factors, such as urbanization, environmental changes, and population growth, have further exacerbated the problem. The increasing number of difficult-to-treat infections underscores the need to preserve existing antibiotics through innovative therapeutic strategies.^15,16^

One of the major drivers of antimicrobial resistance is the extensive use of antibiotics in agriculture, a practice that contributes significantly to disseminating resistant bacteria. According to the World Health Organization (WHO), a substantial proportion of global antibiotic consumption occurs in livestock production, where antibiotics are routinely used for growth promotion and disease prevention. However, studies indicate that 30–90% of these antibiotics are excreted in urine and feces as either partially metabolized compounds or in their active form, allowing them to contaminate soil, water sources, and crops through leaching and runoff. This cycle contributes to the spread of antimicrobial resistance genes in the environment, ultimately posing a risk to human health. Moreover, bioaccumulation of antibiotic residues in the food chain can further exacerbate the resistance crisis, highlighting the need for stricter regulations and alternative disease-control measures in agriculture.^17,18^ The clinical implications of AMR are profound, as increasing numbers of multidrug-resistant (MDR) and extensively drug-resistant (XDR) infections are reported worldwide.^19–21^ The resistance crisis affects bacterial infections and fungal pathogens, which have historically been less concerning but have become significant threats in immunocompromised populations. Since the late 1960s, coinciding with the rise of intensive antibiotic therapies, fungal infections have increased dramatically, leading to severe complications in hospital settings. The uncontrolled surge of resistant bacterial and fungal pathogens necessitates urgent action to develop novel combination therapies that can enhance antimicrobial efficacy and prevent resistance.^22,23^

The significant clinical implications of resistance have drawn interest in studies of resistance in various areas. The widespread resistance of microorganisms is the cause of hundreds of thousands of deaths every year. The most serious problem is the increasing resistance of microorganisms to antibiotics and antifungals.^24,25^ Over the past seven decades, the indiscriminate and extensive use of antibiotics has led to the selection of resistant strains for nearly every antibiotic ever introduced. Resistance was first noted shortly after the introduction of the earliest antimicrobial agents, such as sulfonamides, in the late 1930s.^26,27^ At the beginning of the 20th century, bacterial epidemics were a global and essential cause of mortality. In contrast, fungal infections were almost not considered. However, since the late 1960s, coinciding with the development of antibiotic therapies, there has been a marked increase in microbial infections, which now pose a significant global health threat.^28,29^

The importance of aPDT lies in its ability to circumvent many of the limitations associated with conventional antibiotics. Based on light-induced ROS production, the therapy’s mechanism is effective against many bacteria, fungi, and even biofilm-associated pathogens.^30^ Furthermore, its ability to synergize with antibiotics opens new avenues for combination treatments that enhance therapeutic outcomes and limit resistance evolution.^31^

Despite its potential, several challenges remain, including optimizing treatment parameters, selecting appropriate PS and light sources, and standardizing clinical protocols. Addressing these challenges is critical for the widespread clinical adoption of aPDT as a complementary antimicrobial strategy.

The current state of knowledge suggests that, when used alongside antibiotics, aPDT can improve treatment efficacy, reduce antimicrobial resistance, and enhance bacterial eradication. Several studies highlight its effectiveness against diverse pathogens in different clinical settings, yet critical knowledge gaps remain, particularly in optimizing PS-antibiotic combinations, light dosimetry, and treatment standardization.^5,31,32^ The lack of large-scale clinical trials and regulatory frameworks also presents significant hurdles to widespread adoption. Further research is essential to clarify the mechanisms underlying aPDT-antibiotic synergy, refine therapeutic guidelines, and advance its integration into mainstream antimicrobial therapy.^12,33^ This review aims to synthesize current research and clinical findings on aPDT as an adjunct to antimicrobial therapy, evaluating its mechanisms of action, synergistic effects with antibiotics, and therapeutic outcomes. By providing an in-depth analysis of aPDT’s role in enhancing infection control, this work seeks to guide future research, clinical applications, and policy recommendations in the fight against antimicrobial resistance.

## Methodology

A comprehensive literature search was conducted to gather high-quality evidence regarding aPDT as an adjunctive strategy to antibiotics. The search was performed across multiple scientific databases, including PubMed, Scopus, Web of Science, Google Scholar, and Embase. These databases were selected due to their extensive coverage of peer-reviewed biomedical sciences, microbiology, and antimicrobial research literature. The search was limited to peer-reviewed studies published between 2000 and 2024 to ensure the most relevant and up-to-date findings. Studies were excluded based on the following criteria: articles focusing solely on oncological applications of PDT, without relevance to antimicrobial therapy; studies lacking experimental validation of aPDT-antibiotic synergy; reports without detailed methodologies, such as opinion papers or editorial comments; duplicated studies across multiple databases; clinical trials that did not include microbial resistance analysis; studies that did not specify the type of photosensitizer or irradiation parameters used in aPDT. For each included study, data were extracted from study type (in vitro, in vivo, clinical trials); microbial strains tested; photosensitizers used and their properties; light sources and irradiation parameters; antibiotic combinations and dosing regimens; outcome measures (bacterial reduction, resistance modulation, cytotoxic effects, clinical applicability).

## Summary of Relevant Literature

### Antibiotic Therapy

#### Antimicrobial

In the early 20th century, the medical landscape was radically redefined by the discovery of antibiotics, bioactive molecules synthesized by specific microorganisms that curtail the proliferation of competing bacteria. Before that, infectious diseases such as smallpox, pneumonia, tuberculosis, syphilis, and cholera were among the leading deadly diseases worldwide and kept the average life expectancy below 50 years in industrialized nations.^34^

The paradigm of “selective toxicity,″ a term inextricably linked to Paul Ehrlich’s conceptualization of the “magic bullet,” denotes a targeted pharmacological intervention that effectively eradicates pathogens while minimizing adverse effects on the host organism. This principle was serendipitously validated in 1928 when Alexander Fleming discovered a mold—later classified as *Penicillium notatum*—capable of producing a substance with potent bacteriolytic capabilities. This discovery paved the way for a new era in antimicrobial therapy, culminating in the isolation and mass production of penicillin by Ernst Chain and Howard Florey, ultimately revolutionizing clinical medicine.^35–37^ The widespread deployment of antimicrobial agents ushered in what is now known as the “Golden Age of Antibiotics,″ a period marked by an unprecedented surge in the discovery, refinement, and chemical optimization of various antimicrobial compounds.^1,36^ Notably, spore-forming bacteria played a crucial role in this period, as their derived compounds were systematically modified to enhance their therapeutic potency and broaden their antimicrobial spectrum, enabling the effective treatment of a wide range of bacterial infections.^38,39^

In scholarly discourse, the stratification of antibiotics is based on their distinct chemical architectures, which confer specificity to their mechanisms of action against bacterial targets. Such mechanisms frequently evoke the lock-and-key paradigm, highlighting the exactitude with which those agents engage with bacterial cellular structures. The potency of an antibiotic is contingent upon its bioavailable concentration at the site of infection, the inherent or acquired susceptibility of the bacterial strain, and the presence of any pharmacodynamic interactions—synergistic or antagonistic—concomitantly administered therapeutics.^27,40^

The introduction of antibiotics heralded a transformative epoch in clinical medicine, precipitating a profound decline in mortality attributable to bacterial infections. However, the ascendancy of antibiotic-resistant bacterial strains constitutes a significant impediment, undermining the therapeutic action of these agents. To mitigate the proliferation of resistance, prudence in antibiotic administration must be exercised strictly, conforming to established medical protocols that prescribe appropriate dosages and treatment durations. Such judicious use is paramount to preserving antibiotics’ clinical efficacy and protecting public health.^41^

#### Mechanisms of Action of Antimicrobials

In antimicrobial pharmacotherapy, the specificity of antibiotics (ATBs) arises from their selective interaction with bacterial molecular structures, which are absent in eukaryotic cells. These interactions allow ATBs to exert their effects through distinct mechanisms, which include (i) inhibition of cell wall biosynthesis, (ii) disruption of protein synthesis, (iii) impediment of nucleic acid replication and transcription, (iv) inactivation of critical bacterial enzymes, and (v) compromise of bacterial cell membrane integrity (Figure 1). The mechanism of action of a given antibiotic is directly linked to its chemical class and structure, which defines its efficacy and clinical applications.^42^

**Figure 1:**
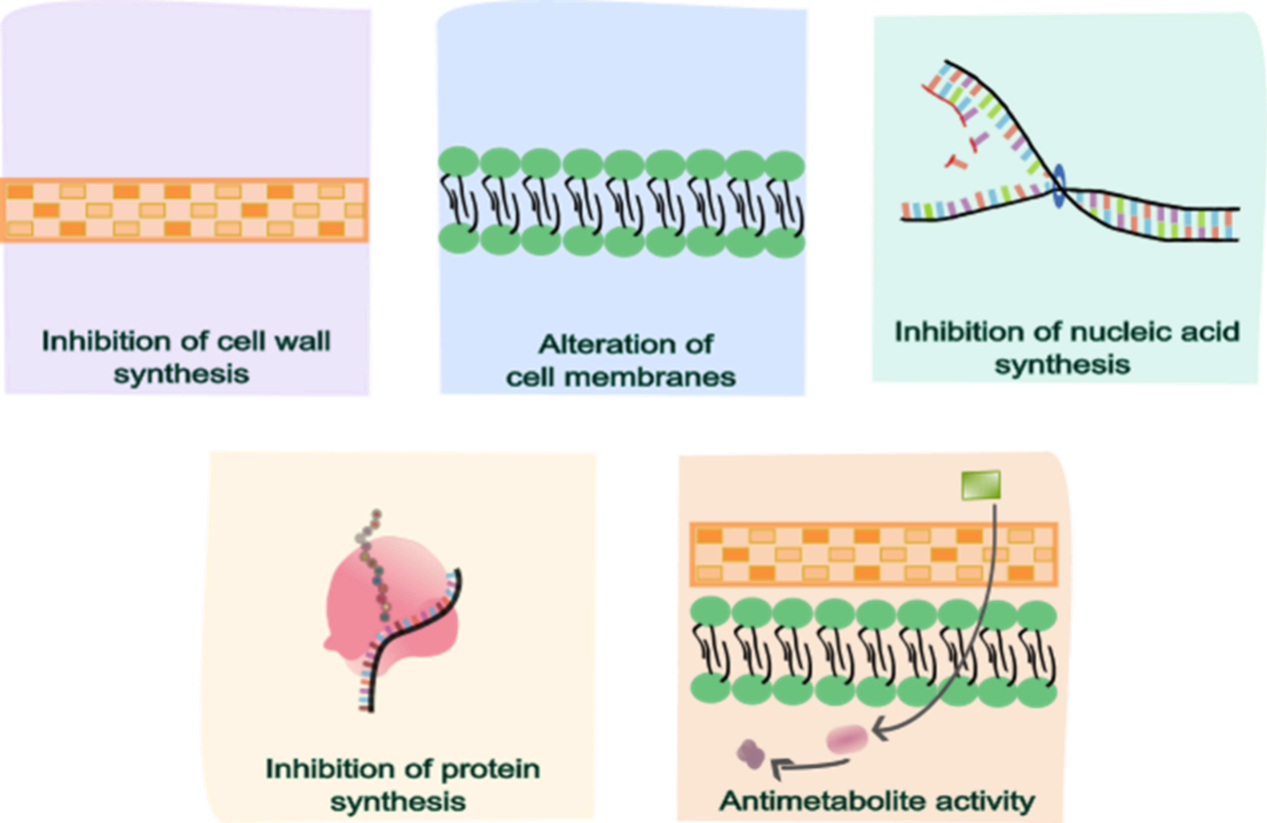
Schematic illustration of bacterial cell targets of antibiotic action, such as inhibition of cell wall synthesis, cell membrane alteration, nucleic acid synthesis, inhibition of protein synthesis, and antimetabolite activity.

Figure 1 illustrates the primary bacterial targets of antibiotic action. β-lactam antibiotics (e.g., penicillins and glycopeptides) disrupt peptidoglycan cross-linking, weakening the bacterial cell wall and inducing osmotic imbalance, leading to lysis and cell death. Polymyxins target the outer membrane of Gram-negative bacteria, disrupting its phospholipid architecture and increasing permeability, while daptomycin integrates into the bacterial membrane, depolarizing it and disrupting ion gradients.^43,44^

Protein synthesis inhibitors act by binding to bacterial ribosomes, disrupting mRNA translation. Aminoglycosides irreversibly bind to the 30S subunit, causing the misincorporation of amino acids into proteins. Tetracyclines block aminoacyl-tRNA from binding to the ribosome, while macrolides target the 50S subunit, preventing peptide elongation. These disruptions compromise bacterial viability.^45–47^

Antibiotics can also target nucleic acids. Rifampicin binds to bacterial RNA polymerase, blocking transcription, while quinolones inhibit topoisomerases, leading to DNA damage and cell death. Sulfonamides, in turn, inhibit folic acid synthesis, impairing nucleic acid production.^48–50^

Regardless of the specific mechanism, all antibiotics aim to inhibit bacterial growth and survival. However, bacterial resistance mechanisms have evolved due to their finite number of action pathways, compromising their efficacy. These mechanisms are discussed in detail in the section on antibiotic resistance.^51^

#### Mechanisms of Antibiotic Resistance

The mechanisms of ATB resistance are related to genetic modifications in receptor biomolecules, which alter their interaction with the ligand, changing the biological response. Those mechanisms may involve alterations in membrane permeability for restricting drug absorption, inhibiting protein synthesis, DNA replication, and RNA polymerization, and causing metabolic and structural modifications in the cell wall.^27,51,52^

The antibiotic resistance of bacteria can be intrinsic, acquired, or adaptive. The inherent properties of the bacteria cause intrinsic resistance. Some bacterial genera or species lack the target site for the specific ATB, or when their structure naturally differs from the ATB’s target, thus making them ineffective. Examples include the glycopeptide resistance of Gram-negative bacteria, which is due to the impermeability of the outer membrane present in their cell envelope.^52,53^

Naturally susceptible bacteria against certain ATBs develop acquired resistance by receiving genetic codes from other bacterial strains through horizontal gene transfer, which includes three main mechanisms, namely, transformation (when free or “naked″ DNA transformation is taken up by other cells in vitro conditions as well as from the environment), transduction (when a bacteriophage transfers DNA between bacteria), and conjugation (when genes are moved horizontally to the recipient cells, leading to sharing of plasmids and transposons between the donor and the recipient cells).^24,54^

Intrinsic or acquired resistance can significantly impact the action of ATB agents, as illustrated in Figure 2.

**Figure 2:**
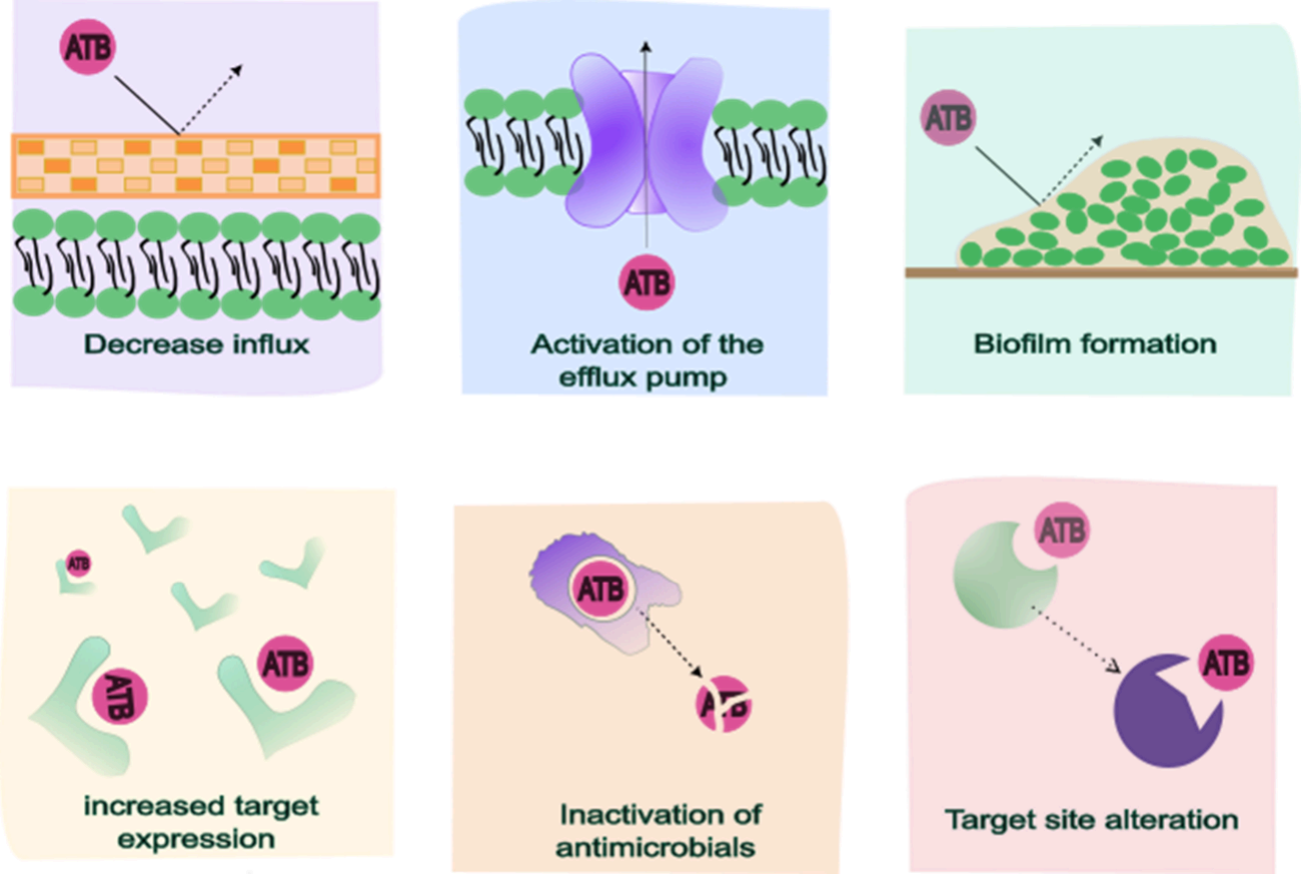
Schematic illustration of the strategies adopted by bacteria to defend themselves against the action of antimicrobial agents (ATBs), including decreasing influx, activation of the efflux pump, biofilm formation, increasing target expression, inactivation of antimicrobials, and alteration of the target site.

Some mechanisms are directly associated with specific classes of ATBs (e.g., ATB inactivation, increased expression of cellular targets, or modification of action sites), and some can affect multiple classes of ATBs, such as cell wall impermeability, increased expression of efflux pumps, and biofilm formation.^55,56^ Resistance mechanisms propagate through artificial selection due to the inefficiency of the ATB in cells with intrinsic or acquired resistance traits. Such cells survive the treatment and become the majority of the microbial population.^34,51,57^

Numerous pathogens worldwide have developed resistance to ATBs, with specific ones considered globally significant for monitoring due to their elevated mortality rates, widespread resistance prevalence, and ease of transmission. These include bacteria from the Enterobacteriaceae family, such as *Escherichia coli* (critical priority), *Staphylococcus aureus*, *Helicobacter pylori* (high priority), and *Streptococcus pneumoniae* (medium priority). Therefore, prioritizing new antimicrobial discoveries, whether in the form of new ATBs or new therapies, is highly encouraged and necessary.^1,14^

##### Modifications of the Antimicrobial Molecule

Bacteria can produce enzymes that inactivate the drug by adding specific chemical moieties to the compound or destroying the molecule, rendering the ATB unable to interact with its target.^24,53^

*Chemical alterations of the ATB*: Gram-positive and Gram-negative bacteria can produce enzymes that cause chemical changes to the antimicrobial molecule. The most frequent biochemical reactions involved include (i) acetylation, (ii) phosphorylation, and (iii) adenylation, which result in a steric hindrance that decreases the ATB avidity for its target.^35,58^

*Destruction of the antibiotic molecule*: β-lactamase enzymes are the most common mechanism in bacterial resistance. They destroy the amide bond of the β-lactam ring, rendering the ATB ineffective. The development of newer generations of β-lactams has systematically been followed by the rapid appearance of enzymes capable of destroying any novel compound, in a process that is a prime example of antibiotic-driven adaptive bacterial evolution.^59,60^

##### Prevent Antibiotic-Target Interaction

Bacteria have developed mechanisms to prevent the antibiotic from reaching its intracellular or periplasmic target by decreasing the uptake of the antimicrobial molecule.^40,51,61^

*Decreased antibiotic penetration/permeability:* For ATBs that have intracellular bacterial targets or, in the case of Gram-negative bacteria, the targets are located in the cytoplasmic membrane. The ATB must penetrate the outer and/or cytoplasmic membrane to exert its effect, an important mechanism in Gram-negative bacteria since it limits the influx of substances from the external environment.^24,58^

*Efflux pumps*: Efflux pumps are an active extruding method of antimicrobial compounds that eliminates ATB from the bacterial cells. The systems may be substrate-specific or have broad substrate specificity. Many classes of efflux pumps have been characterized in Gram-positive and Gram-negative bacteria.^24,35,53,58^

##### Changes and/or Bypass of Target Sites

A common strategy for bacteria to develop antimicrobial resistance is to avoid the action of the ATB by interfering with their target site. Bacteria protect the target (avoiding the ATB reaching its binding site) and modify its site, resulting in decreased affinity for the ATB.^42,62^

*Target protection*: Despite identifying genetic determinants coding for proteins mediating target protection in the bacterial chromosome, the majority of clinically relevant genes involved in this resistance mechanism are carried by mobile genetic elements (MGEs).^24,35,53,58^

*Modification of the target site*: Almost all families of antimicrobial compounds are affected by the insertion of changes to the target site, which is one of the most common mechanisms of ATB resistance. Such changes can be (i) point mutations in the genes encoding the target site, (ii) enzymatic alterations of the binding site, or (iii) replacement or bypass of the original target. Regardless of the type of change, the final effect is always a decrease in the affinity of the ATB for the target site.^51,53^

##### Resistance due to Global Cell Adaptive Processes

Resistance to ATBs can be caused by a global adaptive response in the bacterial cell instead of single changes. Bacteria have devised complex mechanisms to avoid the disruption of pivotal cellular processes such as membrane homeostasis and cell wall synthesis. The development of resistance to vancomycin and daptomycin is the most clinically relevant example of resistance phenotypes resulting from a cell adaptive response to an antibacterial attack. Daptomycin kills the bacterial cell by altering cell membrane homeostasis. The intermediate susceptibility of *Staphylococcus aureus* to vancomycin seems to result from changes that usually involve genes forming part of regulatory systems controlling cell envelope homeostasis.^24,35,53,58^

#### Current Challenges on Microbial Resistance

The number of resistant bacteria and new ones becoming resistant to treatments with all known antibiotics has risen. Few new agents are in the pipeline, requiring the urgent development of new antibiotic classes to avoid major global health tragedies.^63^ The current shortage of effective therapies, lack of successful preventive measures, and existence of only a few new antibiotics require the development of new alternatives for treatments and antimicrobial therapies.^64^

Antibiotic combination therapy involves prescribing two or more antibiotics simultaneously to achieve synergistic activity, which is more beneficial for treating patients. This approach encompasses nanotechnology-based solutions, the application of alternative media, host cytokine responses, computer modeling, and aPDT, as well as the discovery of novel therapeutic strategies. SOS response can be a significant step in the development of drug resistance.^65–68^

Research has recently focused on possible ways to annihilate antibiotic-resistant bacteria without necessarily developing new antibiotics. The main idea is to neutralize microorganisms’ natural resistance defense mechanisms, thus making existing antibiotics more efficient and promoting easy access to more effective ones for treating infectious diseases at a possibly cheaper cost.^35^

Innovative emerging techniques include computational tools that utilize databases to study and predict antimicrobial structures and functions based on genome sequence data. Computational methods have been established and continuously improved to identify novel biosynthetic pathways and implement computational approaches to natural product discovery.^69,70^ Predicating small-molecule products of a wide range of biosynthetic pathways directly from genome sequence data is a daunting challenge. An enormous variety of enzymes synthesizes and tailors natural product scaffolds, leading to countless variations on established chemical structures. From a computational perspective, the problem can be reduced to acquiring a comprehensive training data set that covers such diversity and complexity.^70^ In the long term, enhancing these training sets for algorithms that predict substrate specificity may be necessary by systematically generating large volumes of experimental training data.^69,71^

Predictions of chemical structures directly from genome data would help distinguish known scaffolds from potentially novel ones during a very early stage of dereplication, and training machine learning algorithms with a sufficient quantity of genome data from microbial producers might ultimately lead to reasonably accurate predictions of chemical structures.^69,71^

### Antimicrobial Photodynamic Therapy

#### Photodynamic Therapy

In 1900, the German scientist Otto Warburg, not Oscar Raab, accidentally discovered an association between light and a photosensitive molecule. During an experiment, he exposed a culture of *Paramecium caudatum* containing acridine to white light. He noticed that the cytotoxic effects were more intense when such light was combined with the acridine molecules. Based on that discovery and an understanding of fluorescence, Raab claimed cytotoxicity resulted from energy transfer from light to the photosensitive molecule, which, in turn, converted the fluorescence products into toxic substances for cells, following a mechanism similar to that of chlorophylls.^72^ In the same year, French neurologist Jean Prime reported that the oral administration of eosin to an epilepsy patient caused dermatitis when the patient was exposed to sunlight. It was the first report on the clinical use of the association between light and a photosensitizer in humans. Subsequently, Raab, Jodlbauer, and von Tappeiner demonstrated that a third element, namely oxygen, was necessary for the toxic effects to occur in cells. Such a mechanism of action was then termed photodynamic action.^4,73^

At the beginning of the 20th century, both aPDT and ATB treatment were discovered; however, efforts to treat infectious diseases were primarily focused on finding new ATBs with the advent of penicillin, while the development of PS was limited. Due to increasing resistance to various types of ATBs, the search for effective alternatives became urgent, resulting in renewed interest and advancement in research on PS.^74,75^

aPDT has a wide therapeutic optical window and can be used across the entire spectrum, from blue to infrared. It can be applied across a broad spectrum of light wavelengths, from blue to infrared, to treat superficial and deep tissue infections. The choice of light spectrum and photosensitizer depends on the specific microorganism and the depth of the infection. However, light in the red and infrared spectra region has drawn great medical interest, for (“pois”) light in those spectra reaches deeper tissues and can perform less invasive treatments.^76,77^ aPDT is a promising alternative for treating infectious diseases, especially those caused by pathogens resistant to conventional ATBs.^32^

#### Mechanism of Action of Antimicrobial Photodynamic Therapy

aPDT is a technique that produces ROS that triggers oxidative stress. Cell death occurs when the molecular mechanisms of protection against oxidative agents are inefficient. According to the method, the production of ROS results from the absorption of a photon at a wavelength corresponding to the absorption band of the photosensitizer molecule, typically in the blue, green, or red regions of the electromagnetic spectrum,^73^ as schematized in the Jablonski diagram in Figure 3.

**Figure 3:**
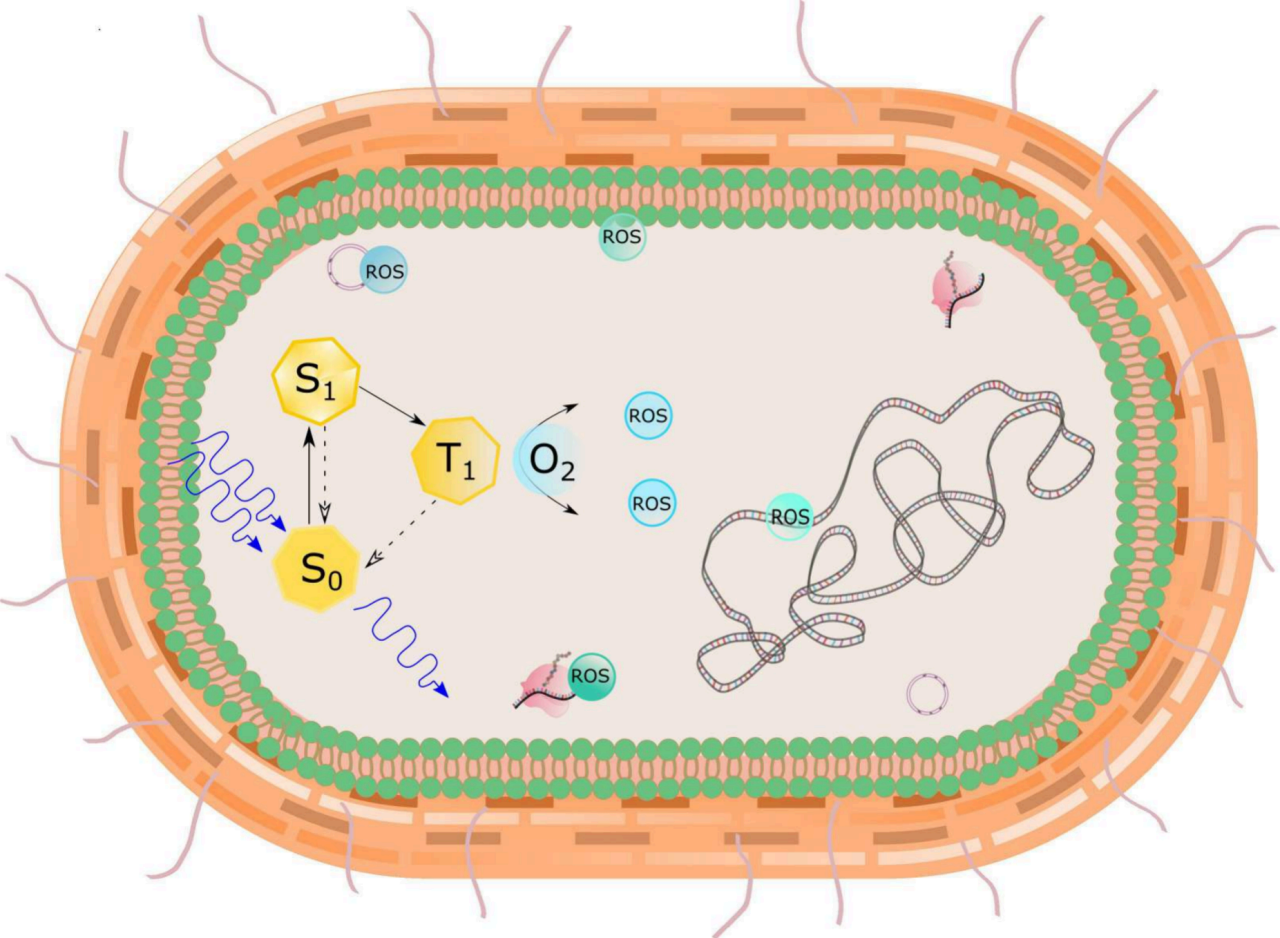
Antimicrobial Photodynamic therapy action mechanism represented by a Jablonski diagram on a bacterial cell. When absorbing a photon with enough energy to promote electrons to more energetic levels, the photosensitizer (PS) internalized in the bacteria transfers the energy or electrons to the oxygen molecule, producing reactive oxygen species (ROS) that will degrade carbohydrates, proteins, lipids, and genetic material. Photosensitizer singlet state (S_0_), singlet excited state (S_1_), triplet excited state (T_1_), and O_2_ molecular oxygen.

The choice of a PS depends on the type of infection. Internal infections require PSs that absorb light at longer wavelengths to promote light penetration through the tissue to the infection site.^73^ Additionally, different PSs may show varying degrees of affinity in the function of the composition of the bacterial cell wall, whether Gram-negative or Gram-positive, which can affect the aPDT effectiveness.^78^

Most PSs are aromatic organic molecules with extensive electronic delocalization. When compared to less delocalized molecules, the energy required to excite electrons from the highest occupied molecular orbital (HOMO) to the lowest unoccupied molecular orbital (LUMO) is lower. Therefore, light in the visible and near-infrared regions of the spectrum has a high probability of being absorbed by the PS, promoting the transition of the ground-state electron (S0) to the excited singlet state (S1), which is unstable and has a short lifetime of approximately nanoseconds. Consequently, several processes occur to stabilize the excited PS (e.g., returning to the initial state (S0) by emitting heat (internal conversion) or light (fluorescence)).^79^ An intriguing possibility for aPDT is the intersystem crossing to the excited triplet state (T_1_), which is less energetic than S_1_, resulting in a higher stability of the PS. Such a nonradiative process involves electron spin inversion, preventing a return to S_0_, since it would violate the Pauli Exclusion Principle because the quantum numbers would be identical to those of its paired electron. With a longer lifetime of the order of microseconds, the excited PS can engage in chemical reactions by two distinct pathways.^74,80^

In type I reactions, electrons (or Hydrogen, H^+^) are transferred to biomolecules such as amino acids, proteins, unsaturated lipids, and nitrogenous bases, which can reduce or oxidize other molecules even after irradiation, initiating redox reactions. Electron transfer to oxygen produces a superoxide radical (O_2._^–^), which undergoes a redox reaction to produce hydrogen peroxide (H_2_O_2_) in the biological medium, catalyzed by the Superoxide Dismutase enzyme (SOD). The hydroxyl radical (OH^–^) can be generated via the Fenton reaction by reducing metal ions such as ferrous ions (Fe^3+^, Fe^2+^), with superoxide acting as the reducing agent. Another possibility of hydroxyl radical generation is the direct reaction of hydrogen peroxide with superoxide, known as the Haber-Weiss reaction.^78,81^

Type II photoprocess produces singlet oxygen (^1^O_2_) through energy transfer from the excited triplet state of the PS to oxygen in the triplet state (^3^O_2_), i.e., its ground state. Singlet oxygen has an unoccupied π∗2p orbital, which makes it highly reactive with electron-rich compounds, a short lifetime (10^–4^–10^–8^ s), and low diffusion (>200 nm). Therefore, its reaction occurs with biomolecules close to the photosensitizer.^79,80^

Type I and Type II reactions co-occur, and their efficiency depends on both the photosensitizer and the availability of oxygen molecules in the medium. However, Type II reactions draw greater interest since the protection mechanisms against oxidative stress do not apply to singlet oxygen. As a result, aPDT is efficient and has a low likelihood of resistance.^32^

#### Photosensitizer

The PS can be molecules, nanoparticles, micelles, and emulsions. Molecules are known as first- and second-generation photosensitizers and broadly divided into two major groups: porphyrinoids and nonporphyrinoids.^82^ Some examples of porphyrinoids used in PDT are porphyrin, chlorin, pheophorbide, and phthalocyanine. On the other hand, PSs such as methylene blue (MB), 83 toluidine blue, eosin MB, hypericin, and curcumin are classified as nonporphyrinoids.^83–86^ The PS characteristics that interfere with the aPDT outcome are partition coefficient, electric charge, optical characteristics, and ROS generation rate.^87–90^

Besides molecules, there are also nanoparticles (NPs),^87–90^ nanomaterials (NMs),^91,92^ and biopolymers,^93^ among others. Among metallic nanoparticles, gold nanoparticles^94,95^ silver,^96,97^ copper, and functionalized metallic nanoparticles^98^ can be mentioned. Those agents can also yield different results in function of their base material, synthesis route, size, electric charge, optical characteristics, and functionalization.^99–105^

The development of new photosensitizers, new applications, and formulations of first- and second-generation photosensitizers has been widely studied and published.^91,95,98,106,107^ Figure 4 shows the growing research development on photosensitizers, photodynamic therapy, and antimicrobial resistance indexed in the Web of Science (Clarivate).

**Figure 4:**
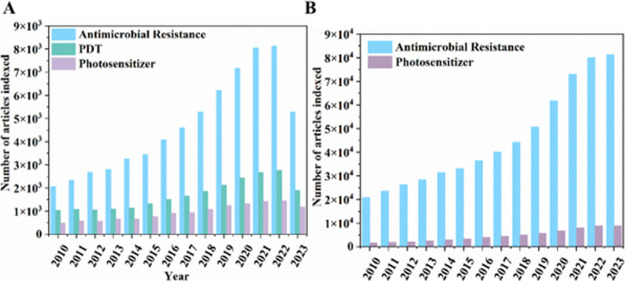
Comparison of publications on antimicrobial resistance, antimicrobial photodynamic therapy, and photosensitizers. (A) Number of articles indexed in the Web of Science Platform. (B) Number of articles on the Scopus platform.

According to the Figure 4, the number of articles on antimicrobial resistance is higher than the subgroups of photodynamic therapy and photosensitizers. The data suggest that traditional aPDT has not yet dominated debates on combating antimicrobial resistance. In this sense, new alternatives, such as using associated therapies like the combination of PDT with antibiotics, can help mitigate the antimicrobial resistance problem.

#### Light Source

In PDT, selecting an appropriate light source plays a fundamental role in determining the efficiency of the treatment since light is a key factor in the photodynamic activity, enabling the creation of reactive oxygen species. Several light sources are available to meet distinct needs, including standard lamps, LEDs, and lasers, with some application differences (e.g., emission spectra, coherence, power, and price). In addition to the type of light source, the photosensitizer’s excitation energy requirements and ability to penetrate tissue and target cells must be considered.^76^

For better technique performance, the light source must fulfill a series of requirements to be able to specify applications. The interaction with the tissue can be complex due to the agents that make up the skin since light can interact with the compound as it can be reflected, refracted, scattered, and absorbed.^108^ Moreover, depending on the application depth, light penetration is considered when choosing a light source. In this regard, the therapeutic window, which encompasses the range of wavelengths capable of inducing therapeutic effects, has been delineated as falling between 600 and 800 nm for nonsuperficial treatments. This range is attributed to the penetration capacity of light at these wavelengths. More energetic wavelengths, such as blue light (∼400 nm), are used for superficial treatments.^109,110^

Standard lamps are often chosen due to their low cost and broad applicability. They are simple, affordable, and offer a broad range of power and wavelength illumination. However, they require optical filtering (UV and NIR radiation), manage thermal effects, experience high energy losses, and producing a nonhomogeneous power distribution.^76,109^

Light-emitting diodes (LEDs) also offer benefits, such as low cost and efficiency in small application areas with reduced thermal effects. They provide more specific emission spectra, determined by the semiconductor’s band gap, covering most of the photosensitizer’s absorption spectra without filtering. Arrays of LEDs can cover larger areas with more homogeneous irradiance and power distribution.^77,111^

For more specific applications, lasers provide exact wavelength targeting with a spectral bandwidth smaller than 0.1 nm and high power output, enabling deep tissue penetration and effective treatment of target cells. They can be coupled with optical fibers for flexible and precise light delivery to specific and internal areas. They produce a localized and coherent beam, which is ideal for targeted therapy, and feature faster modulation speeds than LEDs, hence, more dynamic and responsive treatment protocols. However, they consistently deliver light to smaller areas than LEDs and lamps.^77,109^

Another aspect of light source technologies is how the radiation doses are generated and divided, requiring continuous, intermittent, high, or low power supply. Daylight PDT utilizes natural sunlight as a light source. However, some lamps simulate the spectral emission of the sun and allow indoor application, making them a cost-effective and convenient option for patients, especially for treatments of large or multiple superficial lesions.^30^

Metronomic PDT involves the administration of low and continuous doses of the photosensitizer and light over extended periods, minimizing side effects and potentially enhancing the immune response.^112,113^ Fractionated Photodynamic Therapy (PDT) divides the light dose into several smaller doses, with intervals in between, allowing for the recovery of oxygen levels in tissues. This approach enhances the overall effectiveness of the treatment. By splitting the light dose into two or more smaller doses, fractionated PDT can significantly improve the results in some instances.^114,115^ Each method has its particularities in function of the specific capabilities and applications they offer.

#### Oxidative Stress

Cells acquire essential energy for viability via aerobic respiration, although the process also stands as the primary wellspring of ROS generation within cells, whether prokaryotic or eukaryotic. Molecular oxygen is essential in the respiratory chain since it acts as the final electron acceptor in the respiratory process. However, a small fraction of electrons may interact with molecular oxygen, forming ROS such as superoxide (O_2_^–^) and hydrogen peroxide (H_2_O_2_).^116^

Oxidative stress occurs due to excessive ROS inside the cell and/or when the cell’s antioxidant capacity is limited.^79^ For example, lipid oxidation promoted by H_2_O_2_, particularly in polyunsaturated fatty acids due to their unsaturation and methylene (−CH2−) groups, triggers a free radical chain reaction. This results in the sequential oxidation of lipids through an oxidative cascade.^117^

In response, bacteria produce antioxidant enzymes, such as superoxide dismutase (SOD), catalase, and peroxidase. SOD converts O_2_^–^ into H_2_O_2_, whereas catalase and peroxidase decompose H_2_O_2_ into water and oxygen.^118^ Additionally, bacteria can prevent radical formation through enzymatic control and substrate availability regulation, minimizing the formation of ROS as byproducts. The synthesis of SOD is regulated by SoxRS, which also governs genes encoding DNA repair enzymes and carbon metabolism.^79^ The SoxRS system is generally activated in response to oxidative stress to neutralize free radicals and repair oxidative damage to macromolecules. The OxyR gene also acts as a redox sensor in high levels of H_2_O_2_.^119^ An oxidized OxyR changes its conformation, activating specific DNA sequences of antioxidant enzymes such as SOD, catalase, and peroxidase. It also regulates other genes involved in the oxidative stress response, including those related to DNA repair and redox homeostasis.^116,118^

In aPDT, the singlet oxygen produced in Type II reactions is the most reactive ROS. Cellular metabolism does not produce singlet oxygen; however, it can be generated by endogenous PSs such as flavins and porphyrins in the presence of light.^120^ In photosynthetic cells, it is produced by the excitation of chlorophyll or bacteriochlorophyll pigments.^79^

Type I photodynamic reactions produce ROS, which are commonly secondary products in cellular metabolic processes.^120^ Therefore, intrinsic protection mechanisms combat ROS generated from Type I reactions.^57^ On the other hand, regarding singlet oxygen, the literature reports no enzymes that degrade it. The photooxidative action of aPDT occurs on a large scale inside bacterial cells. Since the expression of oxidative stress control enzymes may be insufficient to prevent the damage caused by ROS,^120^ DT is described as a technique with a low probability of triggering resistance to its mechanism of action. Nonetheless, caution must be taken concerning the elements of the method (e.g., the interplay between the bacterium and the photosensitizer (PS),^121^ These involve a molecular-cell interaction and can potentially be influenced by mechanisms such as efflux pumps.^122^

### Combination of Therapies

The combination of antimicrobial photodynamic therapy (aPDT) with antibiotic treatment represents an auspicious and innovative approach for combating bacterial infections, particularly in light of the alarming rise of antimicrobial resistance (AMR), which threatens to undermine decades of medical progress. This combination strategy leverages the distinct yet complementary mechanisms of action of each modality to achieve enhanced antimicrobial efficacy and reduced likelihood of resistance development. aPDT relies on the activation of a photosensitizer (PS) by a specific wavelength of light in the presence of molecular oxygen, generating reactive oxygen species (ROS) such as singlet oxygen and free radicals. These ROS are cytotoxic and cause irreversible oxidative damage to essential bacterial structures, including membrane lipids, proteins, and nucleic acids, leading to rapid and nonspecific microbial cell death.^78^

When combined with antibiotics, aPDT offers multiple synergistic benefits. One of the primary mechanisms involves the disruption of bacterial membranes, which increases permeability and allows antibiotics to diffuse more easily into bacterial cells.^73^ Additionally, ROS generated during aPDT can impair efflux pump activity, one of the key resistance mechanisms, thereby enhancing intracellular antibiotic accumulation.^120^ Importantly, aPDT is also capable of disrupting and degrading bacterial biofilms, which are notoriously resistant to antibiotics due to their dense extracellular matrix and altered metabolic states. The biofilm disruption enables antibiotics to penetrate more effectively and reach previously protected bacterial populations.^73^ These mechanisms not only boost antibiotic performance but also reduce the minimum inhibitory concentration (MIC) required for bacterial suppression, potentially minimizing systemic toxicity and adverse effects.^73,123^

Recent studies have reinforced these findings, especially against multidrug-resistant (MDR) strains such as *Pseudomonas aeruginosa* and methicillin-resistant *Staphylococcus aureus* (MRSA). For instance, a 2023 study combining aPDT using Photodithazine and gentamicin demonstrated a strong synergistic effect against *P. aeruginosa* , resulting in a significant MIC reduction and enhanced bacterial clearance. Similarly, a 2024 scoping review highlighted the efficacy of aPDT-antibiotic combinations in overcoming resistance mechanisms in *Klebsiella pneumoniae* , using methylene blue and photodithazine as photosensitizers.^124^

Nevertheless, not all combinations yield beneficial effects. Depending on the sequence of administration, bacterial species, and class of antibiotic, certain combinations may result in antagonism, where the photodynamic process alters bacterial physiology in a way that diminishes antibiotic activity. For example, ciprofloxacin combined with certain PSs under sublethal photodynamic conditions has shown variable results, sometimes reducing efficacy due to oxidative stress-induced DNA repair responses or metabolic shifts.^125^

To accurately characterize synergism or antagonism in such combinations, rigorous experimental validation is essential. Mathematical models such as Loewe Additivity and Bliss Independence are commonly used to assess interaction effects. The choice of model is critical, as it influences both data interpretation and clinical applicability. Standardizing these methodologies ensures reproducibility and comparability across studies.^126^

In conclusion, the integration of aPDT with antibiotic therapy presents a robust, multifaceted strategy to enhance antimicrobial outcomes and combat resistant pathogens. The dual-action approach disrupts biofilms, impairs resistance mechanisms, and potentiates antibiotic effects, offering new hope in treating recalcitrant infections. Future clinical translation will depend on the development of optimized treatment protocols, personalized to the type of infection and resistance profile, alongside the continued refinement of analytical models and delivery systems, such as nanocarriers, hydrogels, and implantable light sources.

#### Synergism and Antagonism

The growing threat of antimicrobial resistance (AMR) has outpaced the development of new antibiotics, prompting a search for combination therapies that enhance efficacy, broaden antimicrobial action, reduce side effects, and slow the evolution of resistance. These strategies often involve interactions between antibiotics (ATBs) and adjuvants or alternative treatments, such as antimicrobial photodynamic therapy (aPDT), antimicrobial peptides, bacteriophages, and nanoparticles.^20,127^

For a combination therapy to be effective, its components should ideally exhibit synergism, where the combined effect is greater than the sum of the individual effects. However, an observed enhancement does not always imply synergy, as its definition must follow specific analytical models established in the Saariselkä Agreement in Finland in 1992.^128^ Conversely, antagonism occurs when one treatment diminishes the effectiveness of the other, leading to a weaker overall response.^128,129^ Several mathematical models assess synergy and antagonism, each with different interpretations. Loewe’s additivity model assumes that if two therapies act independently, their combined effect should be the arithmetic sum of their individual effects—any deviation from this suggests synergy or antagonism. However, this model does not account for nonlinear interactions or complex molecular mechanisms.^130,131^

Alternatively, the Bliss independence model defines synergy based on the probability that at least one treatment contributes to an enhanced response. This approach helps quantify the degree of synergistic interaction beyond simple additive effects.^126,132^ Regardless of the methodology used, rigorous experimental validation is necessary to classify therapeutic interactions accurately. The choice of a reference model directly influences the interpretation of synergy, reinforcing the need for standardized analysis methods to ensure consistent and reproducible results.^129^

#### Effect of the Combined Action of aPDT and ATBT

Several studies have investigated the combination of antibiotics (ATBs) and antimicrobial photodynamic therapy (aPDT), testing different microorganisms, photosensitizers (PS), light sources, and antibiotics (Table 1). However, only a minority of these studies have applied standardized methodologies (e.g., checkerboard, post-ATB effects, time-kill assays) or analysis models (e.g., Loewe, Bliss) to correctly classify interactions as synergistic or antagonistic.^12^

**Table 1:** Some Studies on Photodynamic Therapy Combined with Antibiotics, with Details of the Experimental Parameters Used, Such as Type of Photosensitizer, Incubation Time, Wavelength, Light Dose, and Antimicrobial, as well as the Species of Microorganism and the Site of Infection

photosensitizer	incubation time	wavelength (nm)	light dose (J/cm^2^)	antimicrobial	observed effect	microorganism	model/infection site	ref.
5-aminolevulinic acid (ALA)	1.5 h	633–635	100	imipenem, amikacin, clarithromycin, moxifloxacin, rifampicin, ethambutol, levofloxacin	100% cure after 3-month ALA-PDT + antibiotics	*Mycobacterium* spp.	skin infections (clinical study)	^138^
5-aminolevulinic acid (ALA)	3 h	635	100	rifampicin, clarithromycin, minocycline/doxycycline	significant clinical improvement of slow-healing lesions when PDT was added to triple therapy	*M. marinum*	human patient, cutaneous lesion on finger	^149^
5-aminolevulinic acid (ALA)	2 h	635	80 and 160	clarithromycin, moxifloxacin	combined ALA-PDT + antibiotics significantly enhanced bactericidal effect vs antibiotics alone	*Mycobacterium abscessus*	in vitro an in vivo	^150^
chlorin e6-TAT nanoparticle	1 h before irradiation + 4 h postirradiation	635	150	tedizolid	strong synergy vs *P. gingivalis* (CI and Fa analysis)	*Porphyromonas gingivalis*	in vitro + ex vivo (periodontitis in rats)	^151^
CdTe-2.4 quantum dots		480		ceftriaxone, ciprofloxacin, streptomycin, clindamycin, chloramphenicol	synergistic interaction (*S* > 0 in 76.4% of 271 conditions); GIC_50_ values dropped 100-fold	ESBL *K. pneumoniae*, MDR *Salmonella*, *E. coli*	in vitro	^152^
curcumin	15 min	450	10	amoxicillin, erythromycin, gentamicin sulfate	increased susceptibility and reduced resistance after PDI	*S. aureus* and MRSA	in vitro	^7^
curcumin	15 min	450	10 and 20	amoxicillin, erythromycin, gentamicin	up to 32-fold MIC reduction after PDI treatment	*S. aureus*	in vitro	^8^
deuteroporphyrin		360–430 (max 410)	15 and 46	oxacillin, gentamicin, vancomycin, rifampin, fusidic acid	synergy only with oxacillin; no synergy with other antibiotics	*S. aureus* (MSSA, MRSA, h-VISA)	in vitro	^133^
LD4	30 min	650	95	gentamicin	combination group showed significantly greater healing and bacterial clearance	methicillin-resistant *S. aureus* (MRSA)	in vivo (rabbit wound infection model)	^145^
methylene blue	25 min	660	2.8, 5.6, 11.2, 22.4	ciprofloxacin	synergistic PDT + ciprofloxacin effect	*S. aureus*, *E. coli*	in vitro	^135^
methylene blue	15 min	670	10	amoxicillin	mutual uptake boost; up to 8-log MRSA reduction	*S. aureus*, MRSA	in vitro, catheter biofilm, pig skin burn	^153^
methylene blue		625 ± 10	18	gentamicin	synergistic effect vs *S. aureus* and *P. aeruginosa*	*S. aureus*, *P. aeruginosa*	in vitro planktonic and biofilms	^154^
methylene blue	1 h	650	3.6, 7.2, 10.8	tetracycline, chloramphenicol	FICI within additive range (checkerboard)	*S. aureus*	in vitro	^9^
MB-PMX conjugate	10 min	630	6, 144-288	polymyxin B	stronger than MB + PMX-B separately, esp. for gram-negatives	*E. coli, P. aeruginosa; S. aureus*	in vitro, biofilm, porcine skin model	^143^
rose bengal, fullerene	15 min	522	6.4	gentamicin, doxycycline, streptomycin, ciprofloxacin, imipenem, vancomycin, ampicillin, daptomycin, linezolid, tigecycline	synergistic effects with multiple antibiotics	*Enterococcus faecalis*, *E. faecium*	in vitro, biofilm	^155^
rose bengal	30 min	515 nm, 411 nm	20–100	ceftazidime, ciprofloxacin, colistin, doxycycline, gentamycin, imipenem, ampicillin-sulbactam, trimethoprim-sulfamethoxazole, piperacillin-tazobactam.	sublethal aPDI/aBL increased antibiotic susceptibility; synergy observed with increased ROS	*Acinetobacter baumannii*	in vitro	^156^
photodithazine	20 min	660	5, 15, and 25	ciprofloxacin, ceftriaxone, and gentamicin	sequential aPDT after antibiotic; synergy tested via CFU count	*P. aeruginosa*	in vitro	^157^
PPIX-MED	30 min	650	6	ceftriaxone	no synergy for MRSA (FICI = 1), additive for *P. aeruginosa* (FICI = 0.625), *E. coli* (FICI = 0.75); enhanced wound healing	MRSA, *P. aeruginosa*, *E. coli*	in vitro + in vivo (third-degree burns in rats)	^158^
phthalocyanine (IPcZn-Et4+), porphyrin (IP-H-Me2+, Me4+)	1 h	659 (red), 415 (blue)	1.8–5.4 (in vitro), 50 (in vivo)	ciprofloxacin	synergistic effect with IP-H-Me4+ and 5.4 J/cm^2^ in checkerboard assay	*E. coli*	in vitro and in vivo (wound in mice)	^10^
tetracycline + chitosan		315–400 (UVA)		tetracycline	synergy: > 10,000× enhancement vs PDT alone	*C. difficile* (KCTC 5009)	in vitro	^139^
toluidine blue	45	630	5	gentamicin	strong synergistic effect: up to 4.5-log reduction for *S. aureus*, 2.3-log for MDR-*S. aureus*	*S. aureus*, MDR-*S. aureus*	in vitro	^149^
toluidine blue	1 h		5	gentamicin	bactericidal synergy confirmed in vitro/in vivo	*S. aureus* (CMCC 26003, ATCC 43300)	in vitro and in vivo burn model	^146^
ZIF-8-PAA-MB@AgNPs@Van-PEG	4 h		202 mW/cm^2^	vancomycin	superior to individual treatments (PDT/AgNPs)	*E. coli, S. aureus*, MRSA	in vitro and in vivo (mice, endophthalmitis)	^141^

aPDT has demonstrated strong antimicrobial potential, effectively inhibiting microorganisms using only a PS, oxygen, and light. While effective, some pathogens require additional measures for complete eradication, leading to research on complementary approaches that enhance synergy.^12,20,33^ According to Willis et al.^9^ methylene blue-mediated aPDT increased the susceptibility of methicillin-resistant *Staphylococcus aureus* (MRSA) to chloramphenicol and tetracycline. Similarly, Aroso et al.^10^ demonstrated that cationic imidazole-based PS significantly reduced the minimum inhibitory concentration (MIC) of ciprofloxacin in *E. coli* . Studies on curcumin-mediated aPDT further revealed that it could reduce MIC values of amoxicillin and erythromycin while breaking resistance to gentamicin in *S. aureus*, including persister cell populations.^7^

In another study, Iluz et al. investigated the synergy between deuteroporphyrin-based aPDT and oxacillin against *S. aureus*, VISA, h-VISA, and MRSA strains. Light doses of 15 and 46 J/cm^2^ induced synergistic and bactericidal effects, with the highest dose achieving complete bacterial eradication. The MIC of oxacillin was significantly reduced when combined with 34 μM (15 J/cm^2^) and 2 μM (46 J/cm^2^) deuteroporphyrin.^133^

Liu et al. evaluated the synergistic effect of toluidine blue aPDT and gentamicin in vitro and in vivo. The combined use of PDI and gentamicin inhibited the growth and destroyed biofilms of *S. aureus* and MRSA. Liu et al. show that ROS induces oxidative stress and downregulates the expression of AgrA, AgrB, and PSM in the Agr system, resulting in decreased bacterial virulence and infectivity. Similarly, Willis et al. showed that methylene blue aPDT reduced MRSA resistance to chloramphenicol and tetracycline, decreasing MIC values from 32 to 5.03 μg/mL for chloramphenicol and 2.08 to 0.283 μg/mL for tetracycline.^9^ In studies involving curcumin-based aPDT (450 nm irradiation) combined with amoxicillin, erythromycin, or gentamicin, a predominantly synergistic effect was observed when aPDT was applied before antibiotic exposure, as opposed to simultaneous administration. Additionally, this approach homogenized bacterial population responses, improving antibiotic efficacy at lower concentrations.^7,8,11^

Dastgheyb et al. hypothesized that porphyrin could serve as a promising candidate for combination antimicrobial therapies. Given that porphyrin targets bacterial membranes, we reasoned that antibiotics inhibiting protein synthesis would be more likely to act synergistically with porphyrin, in contrast to membrane-active antibiotics that might compete for similar sites of action.^134^ However, not all ATB-aPDT combinations are beneficial. Some pairings, such as gentamicin, vancomycin, rifampicin, fusidic acid, and ceftriaxone with methylene blue aPDT in *S. aureus*, showed no enhanced effects. Antagonism was also reported for vancomycin and rose bengal-mediated aPDT in. *faecium*.^134,135^

Biofilm studies have explored aPDT-ATB synergy, particularly in *S. aureus* and *E. coli* biofilms treated with subinhibitory ciprofloxacin concentrations. The combination proved more effective than monotherapies, regardless of bacterial resistance levels. Likewise, combining aPDT with gentamicin significantly reduced *Pseudomonas aeruginosa* biofilms but had limited effects on *S. aureus* biofilms.^135,136^

A long-term (5 days) methylene blue aPDT and amoxicillin in *S. aureus* found no significant bacterial resistance development. Additionally, oxacillin-resistant MRSA exhibited MIC reduction when treated with deuteroporphyrin-mediated aPDT, suggesting that the PS was not maintained for descendant generations of the treated cells.^133^

In general, these studies highlight the therapeutic potential of aPDT in enhancing ATB efficacy, particularly when applied sequentially rather than simultaneously. However, many studies still lack standardized methodologies and mechanistic insights, limiting broader clinical translation. Future research must focus on establishing validated protocols to ensure that aPDT-ATB combinations effectively combat bacterial resistance without promoting its further evolution.^57,65^

#### Different Strategies for Combining aPDT and Antibiotics

The coadministration and development of combined delivery systems are crucial strategies for the conjugation of antimicrobial photodynamic therapy (aPDT) and antimicrobials, which can enhance treatment efficacy by overcoming the limitations of each therapy individually.

Lu et al., tested the efficacy of aPDT mediated by tricationic fullerene (PS) combined with a suboptimal dose of tobramycin in mice with wounds infected by highly virulent *Pseudomonas aeruginosa* , reporting a synergistic therapeutic effect that cured 60% of the animals.^137^ Similar results were obtained by (Collins and co-workers, who combined another PS from the porphyrin class with the same antimicrobial and achieved more significant bacterial inactivation and a decrease in the minimum inhibitory concentration of tobramycin.^42^ A clinical study applied aPDT mediated by 5-aminolevulinic acid (ALA) combined with standard antimicrobials in four patients with skin infections caused by atypical mycobacteria. All patients showed complete cures after receiving the combined therapy for three months, with no significant side effects reported, thus suggesting the coadministration of aPDT with antimicrobials is a safe and effective method for treating those infections.^138^

In a study conducted by Choi et al., a strategy involving the coadministration of an antimicrobial with aPDT enhanced 10,000 times the efficacy of isolated aPDT, which used tetracycline as both an antimicrobial agent and a photosensitizer against *Clostridium difficile*in vitro, applying chitosan as an auxiliary material to enhance antimicrobial activity. Combining tetracycline with chitosan plus aPDT caused more damage to treated bacteria’s cell membrane and DNA than isolated therapies.^139^

New materials have been developed to potentiate the antimicrobial activity when combined with aPDT. Yin et al. synthesized a quaternized chitosan hydrogel with specific properties for attracting microorganisms.^140^ The hydrogel, containing upconversion nanoparticles and methylene blue, with its cationic macroporous properties, can attract bacterial outer membranes into its structure, leading to their rupture. Besides the mechanical effect, the nanoparticles activate the PS, generating photodynamic reactions that inactivate microorganisms. Combining mechanical effects with aPDT enhances antibacterial action, suggesting a promising new material for treating drug-resistant bacterial infections. Another strategy involved a pH-sensitive metal–organic framework material capable of targeting methylene blue to the infection site in cases of endophthalmitis. The material also carries silver nanoparticles and antimicrobial vancomycin to potentiate the antimicrobial effect of aPDT. It was biocompatible and effective against in vitro and in vivo *S. aureus*, *E. coli* biofilms, and MRSA. After testing the antimicrobial activity of each therapy separately, the authors concluded that combining aPDT with nanoparticles and the antimicrobial along with the material’s properties resulted in a synergistic therapeutic effect.^141^

Conjugating antimicrobials with photosensitizers is another strategy researchers have explored to achieve enhanced effects. Le Guern et al., synthesized a new conjugated molecule that combined cationic porphyrin PS with a polymyxin B derivative. The conjugate showed higher efficiency in eliminating Gram-positive and Gram-negative bacteria during aPDT. It could selectively bind to bacterial cell walls, leading to a more effective treatment.^142^ Ucuncu et al., synthesized a conjugate of polymyxin with methylene blue. The isolated molecule exhibited relative antimicrobial activity, reducing approximately 3 log CFU/mL. However, when illuminated, bacterial inactivation was complete in both planktonic Gram-negative bacteria and biofilms and skin infection models, demonstrating increased efficacy of the combined therapies.^143^

Iluz et al., investigated the synergism between aPDT mediated by porphyrin and conventional antimicrobials in treating infections caused by *S. aureus* and MRSA. No synergistic or antagonistic effect was observed for some tested antimicrobials; however, for oxacillin, the coadministration of both therapies showed a synergistic effect, reducing the MIC in resistant clinical isolates.^133^

Willis, Willis et al. evaluated the photodynamic action of methylene blue (1–4 μM) in combination with antibiotic therapy with chloramphenicol, tetracycline, ampicillin, and kanamycin against different strains of methicillin-resistant *Staphylococcus aureus* (MRSA). The results indicated a reduction in MIC values with the combination of IFD and antibiotics, reducing antimicrobial resistance and restoring sensitivity. PDT slowed the return of drug resistance, allowing >5 passages for the strains to become resistant again. The combination of therapies likely yielded promising results due to the potential binding of methylene blue to the bacterial membrane, especially in regions containing efflux pumps. The generation of ROS may have caused damage to these regions, facilitating the entry and action of antibiotics, significantly slowing the bacterial population of this species.^9^

Liao et al. reported a case in which a 19-year-old patient had a biopsy-proven lesion on his finger caused by the bacterium *Mycobacterium marinum*. Treatment consisted of alternating therapies, starting with 4 months of the antibiotics minocycline, clarithromycin, or rifampin (dual therapy) and, subsequently, 2 months of rifampin, clarithromycin, minocycline, or doxycycline (triple therapy). After 6 months of antibiotic therapy, PDT treatment was initiated, using 20% 5-aminolevulinate (ALA) SP, with light application at a wavelength of 635 nm and an energy dose of 100 J/cm^2^, with three sessions every 15 days. Alternating therapies provided significant improvement in the lesion and treatment of the *M. marinum* infection. The use of PDT can reduce the treatment time for infections and enable better drug effectiveness.^144^

Yin et al. demonstrated the effectiveness of PDT and other drugs in osteomyelitis in rabbits with bone infections caused by MRSA. The PS used was LD4, a porphyrin compound, in different concentrations. Energy doses of 95 J/cm^2^ were applied at a wavelength of 650 nm for 10 min. When combined with medications, the antibiotic gentamicin was injected into animals at a concentration of 0.4 mL per day. The results indicated a reduction of >99.9% in treatments that used a combination of therapies. Histological analyses indicated greater bone tissue regeneration and a lower incidence when light and drugs were applied, in addition to less tissue damage.^145^

Liu et al. sought to evaluate the combined action of the antibiotic gentamicin and PDT using toluidine blue as a FS against the bacterium *S. aureus*, both sensitive and multidrug-resistant strains. The results indicated that the action of ROS was able to reduce the virulence of the microorganism, due to its indirect action on pathogenicity genes (AgrA, AgrB, and PSM). Furthermore, the combination of treatments may have beneficially influenced wound healing in infected mice, due to the reduction of inflammation in the wound region.^146^

APDT and antimicrobial ciprofloxacin were tested on *S. aureus* and *E. coli* cells and biofilms. The best synergistic effect was produced when aPDT was applied before exposure to ciprofloxacin, resulting in a more significant reduction of bacteria and bacterial biofilm than monotherapies.^135^ Soares et al., investigated the effect of applying partial cycles of aPDT on resistant bacteria isolated from patients and bacteria that became resistant in the laboratory, demonstrating that the technique can reverse *S. aureus* resistance to three different antimicrobials. The authors suggested that aPDT combined with antimicrobials might be an important tool for treating bacterial resistance and infections through its oxidative action. The study demonstrated the capacity of aPDT to eliminate bacteria, including resistant strains, and its ability to overcome resistance. When combined with antimicrobials, the technique enhances the action of drugs, potentially restoring their effectiveness, reducing the necessary dose for infection treatment, and minimizing side effects.^7^

As previously discussed, the combined action of antibiotics and PDT can yield better results in the inactivation and treatment of bacterial infections. This can be explained by the combined mechanisms of action of both therapies, in which the oxidizing action of ROS can damage the cellular and metabolic structure of the bacteria, facilitating the entry of drugs. PDT can damage the bacterial cell wall and membrane due to the oxidation of lipids, proteins, and DNA, compromising membrane integrity, which can increase membrane permeability to drugs. The action of ROS on the bacterial membrane can also damage efflux pumps, preventing them from expelling antibiotics and leading to increased drug concentrations in the intracellular environment. The disruption of biofilm protective barriers caused by oxidative damage may allow the antibiotic to enter the matrix and, in addition, the presence of persistent bacteria in biofilms could hinder the action of the drug, since they are less susceptible. However, PDT damage can directly affect these cells, inactivating them.^33,147^

Other mechanisms may also explain the effectiveness of the combination of antibiotic therapy and phototherapy, such as damage to antibiotic-inhibiting enzymes, such as beta-lactamases, preventing them from affecting drug action. PDT can also disrupt bacterial resistance mechanisms, affecting microbial DNA repair and cellular oxidative control. Phototherapy can induce an inflammatory response in the infected organism, activating the immune system, which helps control and eliminate the infection.^148^

As we’ve seen, combining therapies has the potential to improve treatment outcomes for infections, as the application of PDT and the generation of reactive species cause oxidative damage to bacterial cells, affecting their structures and allowing the entry of antibiotics that were previously inaccessible due to the development of resistance mechanisms. Therefore, the combination of PDT and antibiotics may be a potential treatment option due to its high efficacy in inactivating bacteria.

### Therapeutic Perspectives of Combining Therapies

#### Technical Challenges, Limitations, and Safety Concerns Related to the Combined Use of aPDT and Antibiotics

Therapeutic approaches will face challenges and limitations, especially regarding aPDT combined with antibiotic therapy. As discussed elsewhere, the choice of antimicrobial concentrations and the optimization of aPDT parameters such as PS concentration, light wavelength, and light dose to achieve maximum efficacy without damaging surrounding tissues influence the treatment’s synergistic and antagonistic responses.^12,32,159–161^ Limited parameter optimization and the implementation of the treatment in vivo or human models are technical challenges. Those include attention to the depth of light penetration, ensuring adequate light penetration to reach deeper bacterial infections, especially in thick or dense tissues, and uniform light distribution, achieving consistent light exposure across the treatment area to avoid uneven photodynamic effects.^32,33,162,163^

Another limitation associated with aPDT is ensuring the stability and bioavailability of the PS in the body, as some photosensitizers may degrade or lose efficacy depending on the solvent or route of administration.^10,164–167^ Although the mechanism of action of aPDT is via oxidative stress, interaction with the PS is critical; since most PSs are large molecules, they are less uptake into Gram-negative bacteria than Gram-positive ones.^168–171^ This is a key point in the performance of aPDT, as better responses are obtained with good interaction between the pathogen and the PS. At the same time, resistance development is possibly related to the aPDT protocol used. Multiple exposures to the PS can promote a lower uptake of these molecules by the bacteria, which reduces the performance of aPDT but not enough to reduce its effectiveness.^121,172^

Since the combination of treatments systematically affects the body, phototoxicity, and damage to healthy tissues caused by the nonspecific activation of the PS.^78^ In case of unwanted exposure to light, the patient’s post-treatment photosensitization must also be considered.^173^ Validating standardized treatment protocols is critical for successful clinical implementation, ensuring consistent and repeatable results across multiple clinical situations.^5,174^ Given the cost-effectiveness and affordability of combination aPDT and antibiotic treatments and the above-mentioned cautions, administering those medicines must be guaranteed to a large patient population.^66,175^

#### Clinical Applications of Antimicrobial Photodynamic Therapy

The clinical implementation of aPDT depends on several factors, including the photosensitizer (PS) used, incubation time, applied light dose, infection environment, and bacterial characteristics.^6,176–178^

For superficial infections, aPDT alone may be sufficient to control the infection. For example, in the treatment of pharyngotonsillitis, a patient who did not respond to antibiotics after 7 days underwent aPDT with curcumin (0.75 mg/mL) and 450 nm LED light (7 J/cm^2^). After three sessions, complete recovery was observed.^179^ Another study reported a case of recurrent tonsillitis (5–6 episodes/year) in a patient awaiting tonsillectomy. To avoid antibiotic use, aPDT was applied, resulting in the elimination of exudate and hyperemia within 1 week, as well as a reduction in tonsil and crypt size after six months.^180^ These findings reinforce the potential of aPDT as a complementary therapy for inflammatory diseases of the pharynx, significantly reducing bacterial load and promoting tissue healing.^181^ Since this infection site allows easy access to PS application and irradiation, a localized treatment may be preferable over a systemic approach. However, the patient’s medicinal history must be evaluated, as they may have previously received or are currently getting antimicrobial treatment. Understanding the combination of aPDT with antibiotic therapy is therefore critical for its clinical application.

In contrast, difficult-to-reach illnesses, like as pulmonary infections, necessitate tailored PS formulation and dosimetry procedures. Animal model studies have demonstrated the viability of this strategy. For example, hairless mice infected with *Streptococcus pneumoniae* underwent aPDT 2 days postinfection using a 780 nm laser and ICG as the PS. In control groups, bacterial counts ranged from 10^3^ to 10^4^ CFU/mouse, whereas 80% of mice treated with aPDT had no detectable bacteria. The survival rate was monitored for 50 days.^182^ However, when transitioning to larger animal models such as pigs, new challenges were identified, particularly in extracorporeal illumination^183^ and efficient PS delivery. The persistence of residual pathogens may justify the combined administration of antibiotics to ensure complete infection eradication.^184,185^

These examples demonstrate that aPDT holds promise as a therapeutic strategy for both superficial and internal infections. However, monotherapy with aPDT may not be sufficient in some cases, either due to the patient’s medical history or the persistence of pathogenic cells. Therefore, future studies should consider the combined approach with antibiotic therapy as essential for achieving full clinical efficacy.

## Discussion

The growing challenge of antimicrobial resistance has highlighted the urgent need for innovative strategies to enhance the efficacy of existing antibiotics. APDT has emerged as a promising approach, leveraging light-activated photosensitizers (PS) to generate reactive oxygen species (ROS) that damage bacterial cells and increase their susceptibility to antibiotics.

Adaptive resistance is the one to one or more ATBs induced by a specific environmental signal (e.g., growth state, stress, concentrations of ions, pH, nutrient conditions, and subinhibitory levels of ATBs). It is transient, which enables bacteria to revert to the original state once the inducing signal has been removed. It seems to result from modulations in gene expression as a response to environmental changes, possibly due to epigenetic changes.^186–188^ ATBs use different mechanisms against bacteria; consequently, bacteria endlessly adopt methods to overcome the effectivity of the ATBs by using distinct types of mechanisms.^24^ A complete understanding of the mechanisms by which bacteria become resistant to ATBs is of prime importance to designing novel strategies that counter the resistance threat.^58^

ATB resistance is further aggravated by the lack of new antibiotic classes in recent years.^74^ Despite the growing and concerning issue of bacterial resistance, few new ATBs have been discovered. Pharmaceutical companies have shown decreasing interest in developing new ATB classes, primarily due to the complexities and high costs of the research and development processes.^65^ Global efforts and a comprehensive approach to the responsible use of drugs are necessary for driving research and development of new antibiotics.^189^ The continuous and rapid decrease in the effectiveness of available antibiotics in treating common bacterial diseases and a simultaneous decline in the rate of new drug development are global healthcare concerns.^65^

Medical science research has been directed toward preventing AMR and providing broad-spectrum activities. Implementing a thorough antibiotic stewardship program must be prioritized, and it could include educational programs and initiatives targeting all healthcare personnel, patients, family members, and the agricultural sector.^190^ Moreover, assays must identify synthetic and natural-product-based hits compounds specifically for clinically relevant indications. A full suite of expertise in genetics, genomics, microbiology, chemical biology, and biophysics is required.^69^

PDT was first demonstrated as an antimicrobial treatment against drug-resistant infections in the healthcare sector in the early 1990s.^74,191^ Major multidrug-resistant bacteria were found to be susceptible to antimicrobial PDT (aPDT) regardless of their drug resistance profiles.^191–193^ To date, resistance to aPDT has been rarely reported.^191^ More effective aPDT systems are continually being developed; aPDT holds great promise for the treatment of localized infections and for combating AMR.^191^ Antimicrobial photodynamic therapy (aPDT) has been recognized like a fundamental tool in modern therapeutics.^191^ It is due to the expanding versatility of photosensitizers (PSs) and the numerous possibilities to combine aPDT with other antimicrobial treatments to combat localized infections.^191^

Several studies have demonstrated that aPDT can enhance antibiotic susceptibility in resistant strains, potentially reducing antibiotic doses and minimizing side effects.^7–12,20,133–135,146,153^ Furthermore, its broad-spectrum efficacy, ability to circumvent typical antimicrobial resistance mechanisms, and potential to restore the effectiveness of existing antibiotics position aPDT as a valuable adjunctive therapy.^4,120,194^ Despite its promise, significant challenges remain, including optimizing treatment parameters, variability in bacterial responses, and regulatory and logistical hurdles that must be addressed before clinical integration.

The choice of photosensitizer is crucial for the effectiveness of aPDT, as it determines ROS generation efficiency, cellular uptake, and interaction with bacterial structures. Porphyrinoid photosensitizers, such as porphyrins, chlorins, and phthalocyanines, offer strong light absorption in the red and near-infrared spectrum, enhancing tissue penetration and photodynamic efficiency. They exhibit high ROS production and broad antimicrobial activity, but their low selectivity and potential cytotoxicity present limitations.^195^ Nonporphyrinoid photosensitizers, including phenothiazines (methylene blue, toluidine blue) and natural compounds such as curcumin and hypericin, have good absorption in the visible spectrum and can penetrate bacterial biofilms effectively.^110,168,170,196^

However, they are prone to photodegradation and may require controlled-release formulations to improve stability.^169^ Functionalized nanoparticles, including metallic (gold, silver, TiO_2_) and polymer-based nanomaterials, have gained attention for enhancing photosensitizer stability, increasing cellular uptake, and improving treatment selectivity. While these materials show promise, toxicity, metabolism, and large-scale clinical validation concerns need further investigation.^140,177,197–200^

Despite its potential, aPDT faces several technical and clinical challenges that hinder its widespread adoption. One primary limitation is the lack of standardized treatment protocols, as variations in light dosimetry, photosensitizer concentration, and exposure duration affect treatment reproducibility and clinical outcomes.^76,162,201^ Light penetration in deep-seated infections remains a significant hurdle, as most current applications are limited to superficial infections.^108–110^

Strategies like near-infrared light sources and targeted drug delivery systems may help overcome this barrier. The variability in bacterial responses to aPDT is another concern, particularly in Gram-negative bacteria with an outer membrane that limits photosensitizer uptake.^168,170,202^ Moreover, regulatory approval and economic viability pose additional challenges, as the development and commercialization of photosensitizers and specialized light devices require extensive clinical validation and cost-effective manufacturing processes.^5,170^

Advancements in technology offer promising solutions to these challenges. Nanotechnology-based approaches have been explored to enhance the efficiency of aPDT by improving photosensitizer bioavailability, enabling controlled release, and increasing specificity toward bacterial targets.^89,90,203^ Artificial intelligence (AI)-driven dosimetry is another emerging strategy, utilizing machine learning algorithms to predict bacterial responses, optimize light distribution, and personalize treatment based on pathogen susceptibility.^204,205^ Additionally, combining aPDT with antibiotics has shown synergistic effects, potentially reducing the risk of bacterial resistance while enhancing antimicrobial efficacy.^12,33^ Future research should focus on identifying the most effective combinations of photosensitizers, light sources, and antibiotics and optimizing treatment parameters for different bacterial infections.^7,33,135^

## Conclusions

This review showed the promising role of antimicrobial photodynamic therapy (aPDT) as an adjunctive strategy to traditional antibiotic therapy in combatting resistant bacterial infections. The exploration of aPDT’s role alongside antibiotics is in its early stages. Whereas its potential is evident, further research is crucial to transition such a promising experimental approach to a reliable, widely accepted clinical strategy. Future investigations should focus on long-term effects, developing resistance to aPDT, and a deeper understanding of bacterial eradication mechanisms. Addressing these challenges, aPDT combined with antibiotics could emerge as a tool against the ever-growing threat of antibiotic-resistant infections, marking a significant advance in antimicrobial therapy and patient care.
